# A Novel Investigation of an In‐Scanner Alternative to the Cold Pressor Test in Healthy Individuals

**DOI:** 10.1002/hbm.70291

**Published:** 2025-07-25

**Authors:** Sonia Medina, Sam W. Hughes

**Affiliations:** ^1^ Department of Clinical and Biomedical Sciences, Health and Life Sciences University of Exeter Exeter UK

**Keywords:** cold pressor task, fMRI, ICA, pain, seed‐based, spDCM

## Abstract

The cold pressor task (CPT) is widely used to study tonic pain during acute and chronic conditions and is often used as a conditioning stimulus to activate descending pain control systems. However, logistical challenges in magnetic resonance imaging (MRI) limit its application, hindering the understanding of CPT's neural dynamics. To address this, we acquired resting‐state functional MRI (fMRI) data from 30 healthy participants before, during and after immersion in gelled‐cold water, the closest in‐scanner alternative to date to CPT for prolonged stimulation. Participants provided subjective pain intensity ratings after each scan, as well as average pain perceived during noxious stimulation, using a numeric rating scale (NRS). Following fMRI, participants rated their pain continuously during identical tonic noxious stimulation of the contralateral hand using a visual analogue scale (VAS). We employed three complementary methods to examine changes in brain function across fMRI conditions: a data‐driven approach via independent component analysis (ICA), seed‐to‐whole‐brain connectivity analysis with the periaqueductal grey (PAG) as seed and spectral dynamic causal modelling (spDCM) to explore effective connectivity changes across the dorsal anterior cingulate cortex (dACC), anterior insulae (AI), thalamus and PAG. NRS scores were significantly higher following tonic cold compared to baseline and recovery conditions. Continuous VAS reflected sustained mild‐to‐moderate pain over 6 min, with average VAS scores not significantly differing from NRS ratings recorded in the scanner. ICA identified engagement of descending pain control and sensorimotor networks during pain, with the latter persisting during recovery. Seed‐based analysis revealed a disengagement between the PAG and cortical/subcortical regions involved in pain processing, such as the dACC, midcingulate cortex, AI, intraparietal sulcus and precuneus. Finally, spDCM revealed tonic pain neural signature was most likely characterised by top‐down inhibitory and bottom‐up excitatory connections. This study establishes the cold gelled‐water paradigm as a potential in‐scanner alternative to CPT. By uncovering key neural dynamics of CPT, we provide new insights into the brain and brainstem mechanisms of tonic cold pain paradigms routinely used in psychophysical pain studies.

## Graphical abstract

We propose cold gelled‐water paradigm as the closest in‐scanner alternative to the cold pressor task for healthy individuals. Using ICA, seed‐based connectivity and spDCM, we reveal descending pain control engagement, PAG‐cortical functional disengagement and dynamic connectivity changes, offering new insights into the neural mechanisms of tonic cold pain.

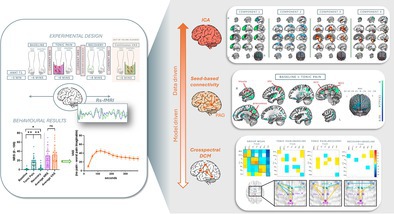

**Summary**

- Immersion in gelled cold water is a reliable in‐scanner substitute for the cold pressor task, enabling prolonged tonic cold pain research in MRI.
- Key neural dynamics, such as PAG‐driven excitatory inputs and AI‐mediated inhibitory control, were identified, providing new insights into cold pain modulation.
- By taking a multimethod approach, including ICA, seed‐based connectivity and DCM, we offer a comprehensive view of the neural networks involved in tonic cold pain, bridging neuroimaging and behavioural research.

## 1 Introduction

The cold pressor task (CPT) is an experimental approach used to induce tonic pain in healthy participants (Duncko et al. 2009; Elias and Ajayi 2019; Huang et al. 2021; Roatta et al. 1998; Watso et al. 2022; Zacny et al. 1996) and chronic pain patients (Johnson and Petrie 1997; Kasch et al. 2005; Nouwen et al. 2006; Oaks et al. 2018; Paccione et al. 2022; Ríos‐León et al. 2024; Stevens et al. 2000; Vaegter et al. 2016). The responses to CPT are typically measured psychophysically, producing mild–moderate pain intensity and unpleasantness ratings (Eccleston 1995). CPT is also routinely used as the most common conditioning stimulus to activate key descending pain modulatory pathways as part of conditioned pain modulation (CPM) paradigms (Kennedy et al. 2016). These CPT responses are therefore likely associated with enhanced bottom‐up and top‐down nociceptive signalling that shape both the pain experience and adaptive response to pain. Thus, detailed assessment of the neural dynamics underlying these responses is essential to decipher how tonic cold pain shapes complex cortical, subcortical and brainstem pain processing pathways.

Functional magnetic resonance imaging (fMRI) represents a useful tool to examine the neural underpinnings of tonic noxious stimulation (Luo et al. 2022). Through measurements of blood oxygen level‐dependent (BOLD) signals, fMRI captures local changes in brain activity during a task, but also allows for dynamic tracking of BOLD fluctuations across prolonged conditions such as tonic pain, an approach called resting state fMRI (rs‐fMRI) (Smitha et al. 2017). Critically, replicating CPT‐like experiments in an MRI environment poses considerable logistical and safety challenges; immersing a hand in water within the scanner bore introduces risks associated with placing conductive fluids near the powerful MRI magnetic field, potentially compromising both safety and data quality (Safety et al. 2020). Consequently, a handful of fMRI studies have used alternative cold stimuli, such as gel pads (Hendriks‐Balk et al. 2020) or aluminium cuffs (Hohenschurz‐Schmidt et al. 2020; Makovac et al. 2020), targeted different body sites like the foot (Hendriks‐Balk et al. 2020; Jarrahi et al. 2018; Richardson et al. 2013), carried out cold noxious stimulation separately from fMRI assessments (Clewett et al. 2013; Grouper et al. 2022) or employed block designs with short lasting periods of hand immersion (La Cesa et al. 2014). These efforts have undoubtedly advanced our understanding of the neural mechanisms underlying tonic cold pain in healthy populations, pointing towards a disruption of descending pain control and autonomic control pathways via modulation of activity in the periaqueductal grey (PAG) (Hohenschurz‐Schmidt et al. 2020; La Cesa et al. 2014; Makovac et al. 2020), amygdala (Clewett et al. 2013; Grouper et al. 2022; Richardson et al. 2013), insular cortex and basal ganglia (King and Carnahan 2019). More specifically, the PAG plays a central role in regulating the body's response to pain by acting as a relay of activation through the descending pain inhibitory pathways (Lau and Vaughan 2014; Mason 1999; Ossipov 2012; Ossipov et al. 2014; Tobaldini et al. 2019). It is therefore reasonable to hypothesise that changes in FC between this region and the rest of the brain may provide some insights into neural mechanisms underpinning the transition between painful and nonpainful states. In addition, converging evidence implicates the engagement of the anterior cingulate cortex (ACC), insula and thalamus during alternative deliveries of cold pain in healthy individuals (Bitar et al. 2020; Duerden and Albanese 2013; La Cesa et al. 2014), making them relevant targets for analysis in tonic cold pain paradigms. Nevertheless, experimental setups that deviate from traditional CPT paradigms inevitably limit the generalisability of the results, hampering our ability to understand brain mechanisms underlying results from the psychophysics literature. Furthermore, the methodological choices to analyse fMRI data across the aforementioned studies varied greatly, ranging from block designs to rs‐fMRI, from whole brain exploratory results to seed‐based analyses constrained to a few regions of interest, from BOLD fMRI to arterial spin labelling fMRI, from 3 to 7 T MRI scanners. This absence of methodological and analytical consistency further impacts the comparability, generalisability and interpretability of the results.

Lapotka et al. (2017) developed an MRI‐compatible alternative to the cold pressor test by adding a thickening agent to the water, creating a gel‐like substance. This method, used to successfully induce experimental pain in women with chronic pelvic pain, demonstrated BOLD activation in pain‐processing brain areas within an evoked‐responses fMRI block design. However, the impact of prolonged tonic cold noxious stimulation through gelled‐cold water hand immersion on brain dynamics during rest in healthy participants remains unexplored.

In this study, we examined the effects of hand immersion in noxious gelled‐cold water on rs‐fMRI dynamics in 30 healthy participants, measuring BOLD responses during 6 min at baseline, during tonic pain and in recovery. We employed three complementary data analysis methods: a data‐driven, fully exploratory independent component analysis (ICA), a seed‐based connectivity analysis and a model‐driven approach using spectral dynamic causal modelling (spDCM). We proposed that this multimethod approach could allow us to maximise interpretability by integrating insights from different analytical perspectives. Through this strategy, we not only provide a robust account of the neural dynamics associated with tonic cold pain but also offer a framework for future studies to understand how methodological choices impact the interpretation of rs‐fMRI data in pain research.

## 2 Materials and Methods

### 2.1 Experimental Subjects

Thirty healthy, pain‐free participants were recruited for this study (18 female; mean age = 29 years, SD = 10). One participant was excluded from data analysis for not completing the MRI session due to high anxiety during scanning. All participants had no history of neurological or psychiatric conditions, no history of acute migraines or concussion resulting in loss of consciousness and no history of dizziness or motion sickness. Additional exclusion criteria included history of substance or alcohol abuse, inability to lie still within the MRI scanner environment, ongoing use at the time of the prescreening of psychoactive medications, such as selective serotonin reuptake inhibitors, serotonin‐noradrenaline reuptake inhibitors, stimulants, antipsychotics, anticonvulsants or benzodiazepines, as well as any medication known to affect temperature sensitivity (e.g., paracetamol, nonsteroidal anti‐inflammatory drugs), endogenous analgesia or cardiovascular function, inability to understand instructions in English and presence of any additional MRI contraindication (e.g., presence of metal in body, pacemaker, pregnancy, etc.). As a precautionary measure, 24 h prior to their visit, participants were instructed to abstain from alcohol or any recreational drugs, to abstain from painkillers or paracetamol for 12 h before their visit and to limit themselves to a maximum of one caffeinated drink in the morning, and to abstain from caffeine for at least 4 h prior to their visit, in order to reduce the potential acute vasoconstrictive and neurostimulant effects of caffeine during scanning. This approach aimed to balance physiological control with ecological validity by allowing participants to maintain their usual morning routines and avoid introducing variability due to caffeine withdrawal. At the beginning of each visit, participants were asked to confirm adherence to these guidelines. If any guidelines were not followed, the visit was immediately terminated and rescheduled. Use of nonpsychoactive or supplements that were taken consistently across visits, such as contraceptives, antihistamines or dietary supplements, was permitted to reflect typical variation in the healthy population. All participants included in the final sample reported no history of recreational or prescribed psychoactive substance misuse. Participants provided written informed consent at a different visit. The experiment was approved by the Health Research Authority and Health and Care Research Wales ethics committee (Ethics reference: 22/HRA/4672). Participants were given the opportunity to withdraw from the study at any point.

### 2.2 Procedure

**FIGURE 1:**
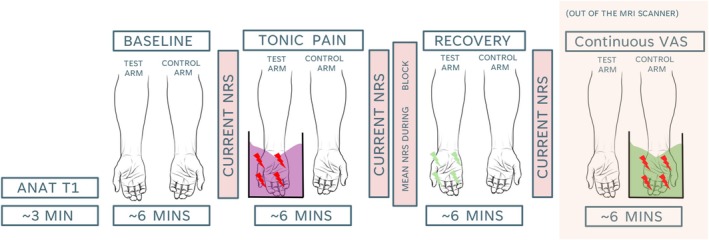
Experimental design. Participants provided their current general perceived levels of pain in a numerical rating scale (NRS) from 0 (‘no pain’) to 100 (‘worst pain imaginable’) following each functional scan. Following the tonic pain scan, participants also provided the average perceived pain during tonic cold stimulation in an NRS. During the tonic pain condition, all participants immersed their right hand in a gelled‐cold water tub. Immediately following fMRI scanning, participants immersed their left hand in a gelled‐cold water tub and provided continuous pain intensity ratings in a visual analogue scale (VAS), consisting of a horizontal line anchored from 0 (‘no pain’) to 100 (‘worst pain imaginable’).

Participants attended five sessions in total (Sessions 1–4 reported elsewhere) and one single MRI session. MRI scanning took place at the Mireille Gillings Neuroimaging Centre facility in Exeter. At the beginning of the MRI session, compliance with lifestyle guidelines and MRI eligibility were assessed. Participants also completed the state‐specific component of the State Trait Anxiety Inventory (Spielberger et al. 1971) to assess participants' anxiety levels on the day. Next, participants underwent localiser and structural scans, followed by three resting state functional MRI (rs‐fMRI) scans, each lasting approximately 6 min (Figure 1). The first rs‐fMRI scan was a *Baseline*, just aimed to assess markers of brain function at rest in the absence of additional stimulation. The second scan represented the *Tonic Pain* condition; before the scan began, a cold gel tub was placed on the participants' stomach over a plastic sheet, in order to avoid cooling the participants' skin. Participants were instructed to fully submerge their right hand in the gel as soon as scanning began and keep it still inside the gel for the duration of the scan. Once the scan terminated, participants were instructed to take their hands out of the tub, and the experimenter entered the scanner room to remove the tub. The final scan mimicked the baseline scan and was aimed at assessing brain function during tonic pain *Recovery*. At the end of each scan, participants answered the question ‘in how much pain are you right now?’ with a numerical rating from 0 (no pain) to 100 (worst pain imaginable). In addition, following the Tonic Pain scan, participants reported their perceived average pain during tonic cold stimulation with numerical rating from 0 to 100. During each rs‐fMRI scan, participants were instructed to lie still with their eyes open and focus on a white fixation cross on a black background displayed at the centre of the screen in front of them. To further understand the temporal dynamics of participants tonic pain throughout the Tonic Pain condition, participants were presented with the same tonic cold stimulation following fMRI on their left hand, in order to avoid carry over effects on their right hand, and they were instructed to provide continuous subjective pain ratings across 6 min on a computerised visual analogue scale (VAS) (Carlsson 1983) anchored with ‘no pain’ and ‘worst pain imaginable’.

### 2.3 Materials and Measures

#### 2.3.1 Cold Gel

The method to deliver noxious tonic cold stimulation followed the strategy implemented by Lapotka et al. (2017). The gel was a mixture of 300 g of cornstarch, 2 L of water and 100 g of salt, mixed in a 2‐L standard, rectangular plastic food container. A pilot testing demonstrated that the temperatures employed by Lapotka et al. (2017) for testing (< 1°C) were not bearable for most participants included in pilot testing for the full duration of the scan; therefore, the tubs were kept in a fridge set to 5°C for at least 24 h before each scanning visit, allowing for tonic stimulation that was bearable for 6 min while eliciting different levels of tonic pain. In order to keep the initial temperature as constant as possible, tubs were taken out of the fridge up to 2 min before they were entered into the scanner room or presented to participants after scanning. During pilot testing, the initial temperature at the time of hand immersion was consistently measured at below 6°C. After 6 min of immersion, the gel temperature typically rose by 2°C–3°C due to heat exchange with the hand. Therefore, fresh tubs were presented on each hand within each session.

#### 2.3.2 MRI Data Acquisition

Data were collected using a 3 T Siemens Prisma MRI scanner equipped with a 64‐channel coil. High‐resolution T1‐weighted structural images were acquired for all participants (repetition time (TR) = 2100 ms; echo time (TE) = 2.26 ms; voxel size = 1 × 1 × 1 mm). For fMRI, we employed a multiecho, multiband (MB) echo planar imaging (EPI) acquisition sequence with the following parameters: TR = 1500 ms; TE1 = 11 ms; TE2 = 27.25 ms; TE3 = 43.5 ms; TE4 = 59.75 ms; MB acceleration factor = 3; 51 slices with a thickness of 2.60 mm; voxel size = 2.6 × 2.6 × 2.6 mm; field of view = 220 mm^2^; flip angle = 77°; 229 volumes; and total scanning time = ~6 min. Slices were acquired in interleaved order. To perform field map distortion corrections, a separate field map scan was acquired at the end of each functional scan (TR = 590 ms; TE1 = 4.92 ms, TE2 = 7.38 ms; 51 slices with a thickness of 2.60 mm; voxel size = 2.6 × 2.6 × 2.6 mm; field of view 220 mm^2^, flip angle 46°; interleaved acquisition).

#### 2.3.3 MRI Preprocessing

Following an initial visual inspection of the data for any abnormalities and artefacts, preprocessing of the fMRI involved several steps. First, motion correction was performed using MCFLIRT (Saccà et al. 2018) from FSL (Jenkinson et al. 2012) to correct for head movements within the fMRI time series. Field map correction was then applied using the FUGUE command in FSL FEAT to address geometric distortions caused by magnetic field inhomogeneities. The optimal combination of echoes and denoising was achieved using the open‐source tool TEDANA (DuPre et al. 2021), which employs principal component analysis (PCA) (Viviani et al. 2005) and independent component analysis (ICA) (Steel et al. 2022) to separate and remove noise from multiecho fMRI data. A visual inspection of each component was carried out to ensure correct noise/signal classification. White matter (WM) and cerebrospinal fluid (CSF) signal regression was then applied, with WM and CSF masks obtained from optimal segmentation of T1‐weighted scans using the DARTEL tool on SPM12 (https://www.fil.ion.ucl.ac.uk/spm/doc/manual.pdf), which were previously coregistered to each functional scan separately using FSL FLIRT. The resulting denoised data underwent high‐pass filtering with a cut‐off frequency of 0.005 Hz to remove low‐frequency drifts. Given the relatively short time series (~6 min), we opted for excluding a band‐pass filtering step, as it could potentially introduce artefacts or attenuate meaningful signals at the edges of the band (Hallquist et al. 2013). Normalisation to Montreal Neurological Institute (MNI) space was achieved in two steps: first, coregistered and segmented grey matter (GM) masks (calculated separately for each run) were normalised to MNI space (2 mm isotropic voxels) applying linear and nonlinear warping (using FLIRT and FNIRT, respectively). Next, the resulting warping parameters were applied to the functional images. Finally, spatial smoothing was performed with a 5 mm FWHM Gaussian kernel to enhance the signal‐to‐noise ratio.

### 2.4 Data Analysis

#### 2.4.1 Self‐Report Data Analysis

In order to assess changes in subjective perceived pain across conditions, numerical rate scale (NRS) measures obtained at the end of each scan were compared via means of a one‐way repeated measures analysis of variance (ANOVA) with condition (baseline, tonic pain, recovery) as a within‐subjects factor. For post hoc pairwise comparisons, Sidak–Holm correction was applied. In order to test whether the average NRS reported following tonic cold in the scanner was comparable to the average pain intensity perceived on the left hand outside of the scanner, a paired‐samples t‐test was conducted between average NRS and average VAS (0 = no pain at all; 100 = worst pain imaginable) for each person.

#### 2.4.2 Independent Component Analysis (ICA)

In order to explore whole‐brain resting state networks arising at the group level across rs‐fMRI conditions, we conducted ICA analysis. ICA identifies a number previously defined by the researcher of spatially independent patterns of brain activity, known as components, which correspond to functionally connected brain networks (Damoiseaux et al. 2006; Schöpf et al. 2010). Each component consists of a time course and a spatial map, where the time course represents the temporal dynamics of the network's activity, and the spatial map shows the brain regions involved in that network. While seed‐based connectivity requires the definition of at least one predefined region of interest (the seed), ICA is a data‐driven approach that can detect multiple networks simultaneously without prior assumptions (Yang et al. 2020). We conducted ICA within each condition using the MELODIC tool implemented in FSL (Beckmann and Smith 2004), setting the number of components to 10 to ensure the focus on large‐scale networks (Abou‐Elseoud et al. 2010). Subsequent dual regression analysis was performed to relate the individual subject data back to the group‐level components identified in the ICA analysis. Finally, the group‐level significance of subject‐specific spatial maps was assessed using randomise (Winkler et al. 2014) with threshold‐free cluster enhancement (TFCE; 5000 permutations, *p* < 0.05).

#### 2.4.3 Seed‐Based Functional Connectivity (FC) Analysis

We explored which parts of the brain exhibited BOLD signals that covaried with our predefined seed region, namely PAG, in order to examine which brain networks were associated with PAG activity across conditions. Seed‐based FC analyses were performed in FSL FEAT. For each participant, FC maps were generated for each condition (Baseline, Tonic Pain, Recovery) using the PAG as the seed region. The PAG anatomical binary mask was sourced from the Brainstem Navigator toolkit (Bianciardi 2021) and resampled to 2 mm isotropic voxels.

FC was assessed by correlating the time series from the seed with time series from all other brain voxels. At the group level, we evaluated differences in FC across conditions using a repeated‐measures design within a general linear model (GLM), including condition‐specific as well as subject‐specific explanatory variables (EVs). Our contrast of interest included pairwise comparisons across sessions (baseline vs. tonic pain, tonic pain vs. recovery and baseline vs. recovery). Finally, to further explore the relationship between PAG connectivity and pain perception, we computed correlation between the FC maps obtained during the tonic pain condition and participants' average NRS within a separate GLM. For all group analyses, significance was assessed using Randomise (Winkler et al. 2014) with TFCE correction (5000 permutations, *p* < 0.05).

#### 2.4.4 Effective Connectivity Analysis

Finally, we performed cross‐spectral dynamic causal modelling (spDCM) to determine whether the patterns of effective connectivity (i.e., the excitatory or inhibitory influence of one brain region on another) within an a priori defined network could account for variability in BOLD signal fluctuations across rs‐fMRI conditions. SpDCM utilises a linear random differential equation:

$$\frac{\mathrm{dx}\left(t\right)}{\mathrm{dt}}=\mathrm{Ax}\left(t\right)+v\left(t\right)$$

where *x*(*t*) denotes the neuronal state vector, *A* represents the matrix of effective connectivity and *v*(*t*) captures stochastic endogenous fluctuations. This method focuses on modelling the cross‐spectral density *G*_xy_(*ω*), derived from the Fourier transform of the cross‐covariance function, to capture frequency‐domain covariance between neuronal variables. By fitting this model to the observed cross‐spectral density, we assessed how effective connectivity explains variations in BOLD fluctuations. For an in‐depth explanation of spDCM, see Novelli et al. (2024).

Regions of interest (ROIs) were selected according to key loci of the descending pain control pathway, namely, the dorsal anterior cingulate cortex (dACC), the anterior insula (AI) and the thalamus and the PAG. ROIs were defined using spherical masks based on MNI coordinates obtained from probabilistic maps in the Brainnetome Atlas (Fan et al. 2016). Specific map labels, MNI coordinates and radius sizes are detailed in the Supporting Information and Medina and Hughes (2025).

SpDCM analyses were conducted in SPM12 (Ashburner et al. 2014). For each participant and fMRI run, a fully connected model was described, where all the possible connections across ROIs were switched on. Model parameters were then estimated via variational Laplace inversion, using the default probability densities provided by SPM12. Parameter estimation (i.e., effective connectivity strengths) was performed at the group level within a parametric empirical Bayes (PEB) framework (Zeidman et al. 2019). PEB is a hierarchical Bayesian modelling approach that estimates group‐level effects on connectivity parameters by pooling information across individuals, while accounting for between‐subject variability and uncertainty in individual parameter estimates. This method improves robustness and sensitivity when modelling effective connectivity across repeated measures. We adopted the same approach described in Hidalgo‐Lopez et al. (2021); first, a PEB design matrix including subject‐specific regressors in addition to condition‐specific regressors, to account for the repeated measures nature of the design, was constructed. However, this full model yielded lower model evidence, as determined by the free energy approximation to the log model evidence, compared to a simpler model including only condition‐specific regressors. In line with established PEB practice, we selected the simpler model for final analysis, as it provided a better balance of explanatory power and model complexity. Thus, to examine differences in effective connectivity within our model across conditions, our final model included 4 regressors: the group mean, the difference between baseline and tonic pain conditions, the difference between tonic pain and recovery conditions and the difference between baseline and recovery conditions. For each regressor, only resulting connections with a posterior probability (the probability of existing) greater than 0.75 are reported (Figure 6).

Finally, we validated our model via leave‐one‐out cross‐validation (LOOVC) implemented in SPM12 (spm_dcm_loo.m). For LOOCV, we only included connections where associated parameters showed a *pp* > 0.90 for at least one condition difference, in order to maximise the accuracy with which these connections predicted the conditions.

## 3 Results

### 3.1 Subjective Perceived Pain

**FIGURE 2:**
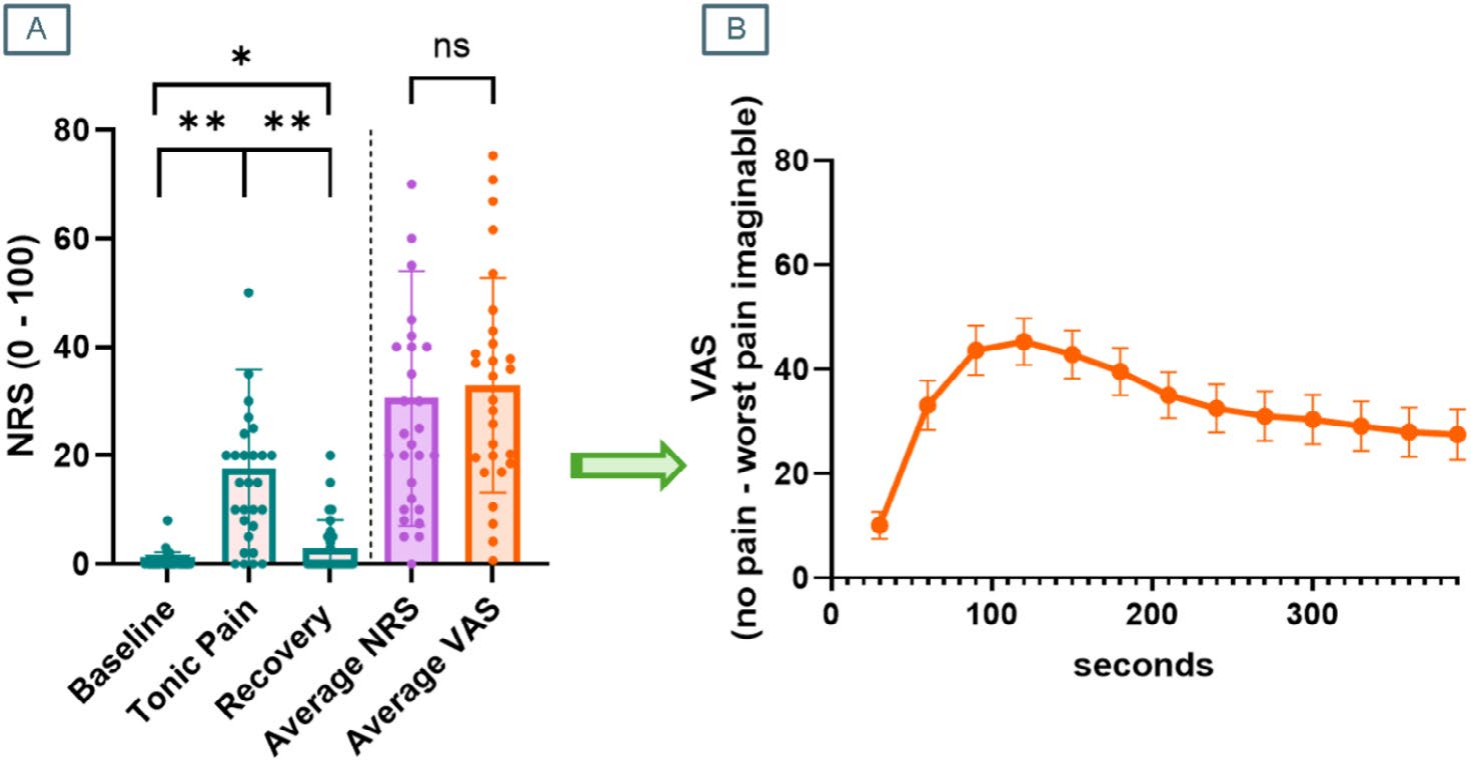
Summary of behavioural results. (A) ‘Current pain’ NRS scores were significantly higher following the Tonic Pain condition than on the other two. Average NRS scores about perceived pain intensity estimated by participants during the Tonic Pain scan did not differ from average VAS provided outside of the scanner for tonic cold stimulation on the opposite hand. (B) Average continuous VAS ratings for 30‐s bins across 6 min. Error bars represent standard error of the mean (SEM).

One participant did not complete the MRI session due to anxiety inside the scanner. The final sample was *N* = 29. One‐way ANOVA indicated a main effect of Condition (*F*_(2,56)_ = 24.48, *p* < 0.001). Pairwise post hoc comparisons indicated that reported pain was significantly higher following the tonic pain scan (mean (SD) = 17.59 (18.23)) than on the other two (baseline mean (SD) = 0.51 (1.61); recovery mean (SD) = 2.96 (5.15)). Perceived pain following the Recovery scan was also significantly greater than in the Baseline scan (Figure 2).

Software for continuous VAS data from one participant failed, and therefore paired‐samples t‐test included a total *N* = 28. There was no significant differences between average NRS (mean (SD) = 28.41 (20.89)) and computed average VAS (mean (SD) = 32.92 (19.75)).

### 3.2 ICA Results

**FIGURE 3:**
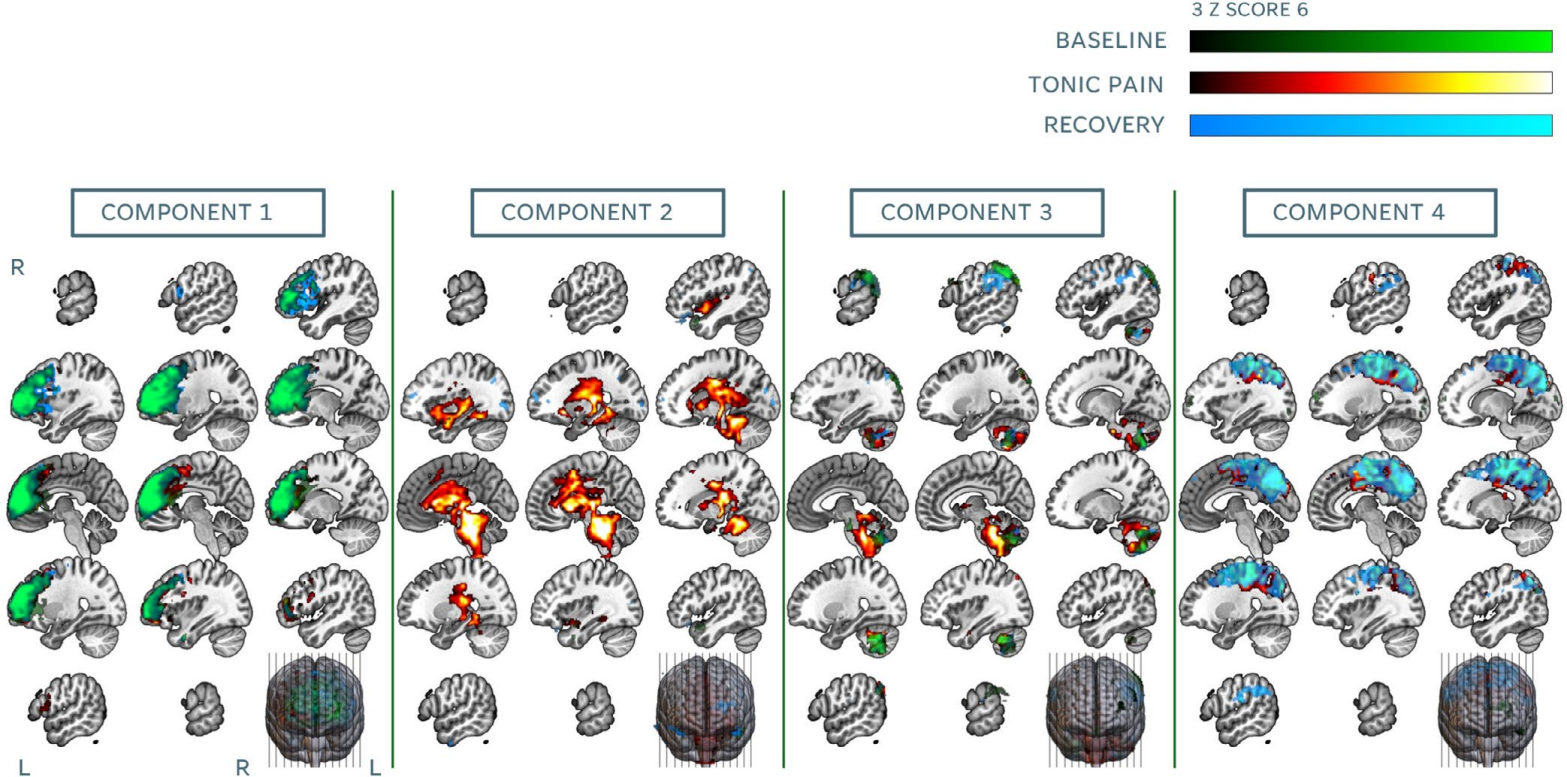
ICA results from components 1–4. Functional networks identified across each condition at group level (green = baseline, red = tonic pain, blue = recovery). First four components were identified by researchers as the ones capturing variability of interest and not artefactual. L = left; R = right.

**FIGURE 4:**
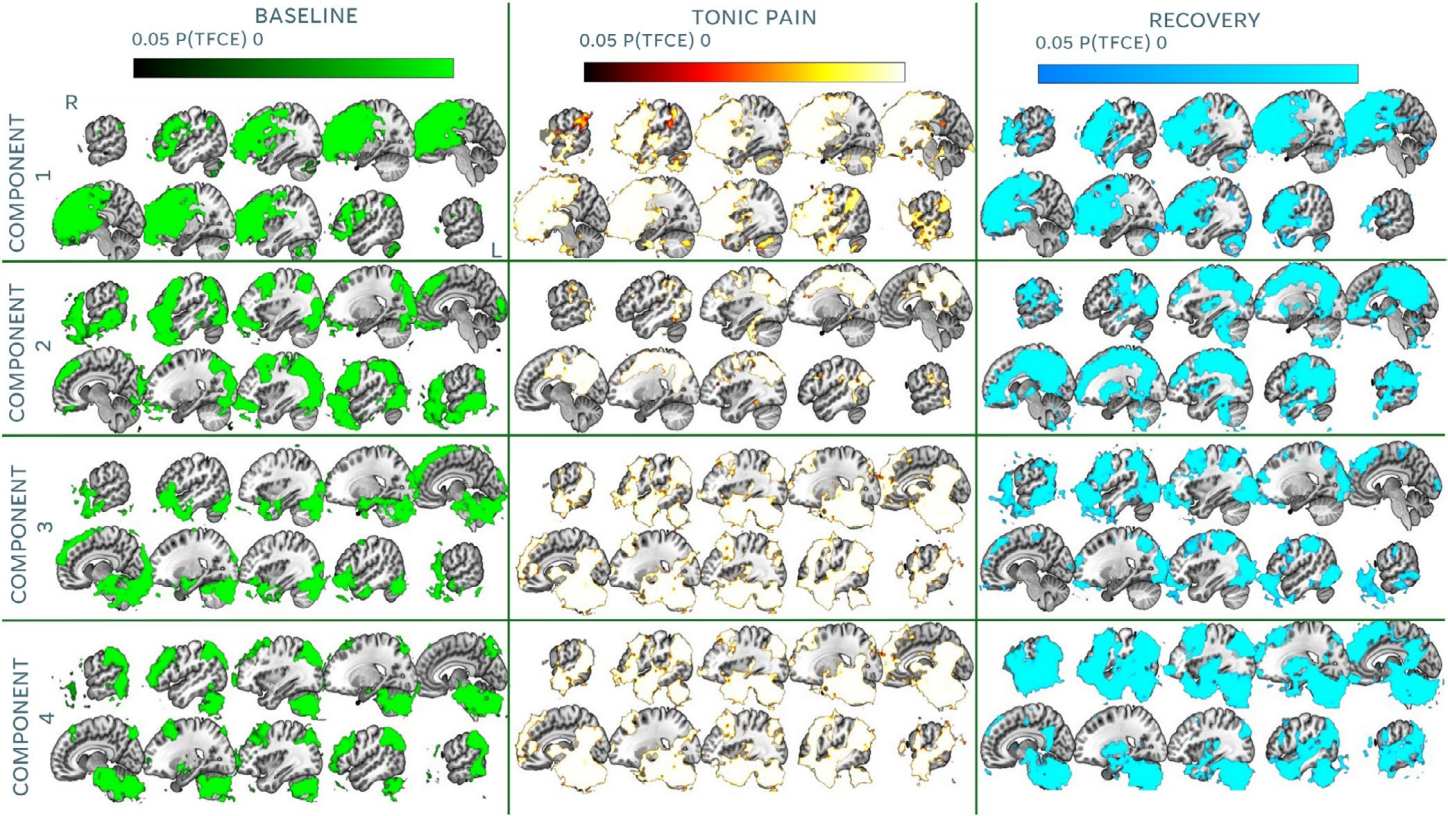
Dual regression results. All maps reflect significant extension of group ICA components at alpha 0.05. Sagittal slices displayed at MNI coordinates (from right to left): 62, 50, 36, 22, 8, −6, −20, −34, −48, −62. TFCE = threshold‐free cluster enhancement. MNI = Montreal Neurological Institute. L = left; R = right.

Following ICA, we focused the analysis on Components 1–4, which demonstrated distinct spatial patterns relevant to our conditions of interest (Figure 3). Component 1 overlapped considerably across the three conditions and corresponded with the frontal part of the default mode network (DMN) (Menon 2023; Raichle 2015). Component 2 involved sensorimotor regions only during tonic pain and recovery runs, and very reduced areas of frontal and occipital cortices during the baseline run. Component 3 was mostly evident during the tonic pain condition, with activity extending across areas involved in ascending and descending pain control, such as dACC, anterior and posterior insulae, basal ganglia, thalamus, midbrain and most of the brainstem. Component 4 involved cerebellar areas overlapping across conditions, extended to lower parts of the brainstem during tonic pain, and included parietal and occipital clusters during baseline and recovery runs. Components 5–10 were considered to be associated with artefactual BOLD activity as well as motion‐related fluctuations on the edges of the brain and can be found in the Supporting Information B. Following dual regression, TFCE‐corrected Z maps indicated that components were statistically significant at the group level across conditions (Figure 4).

### 3.3 Seed‐Based FC Results

**FIGURE 5:**
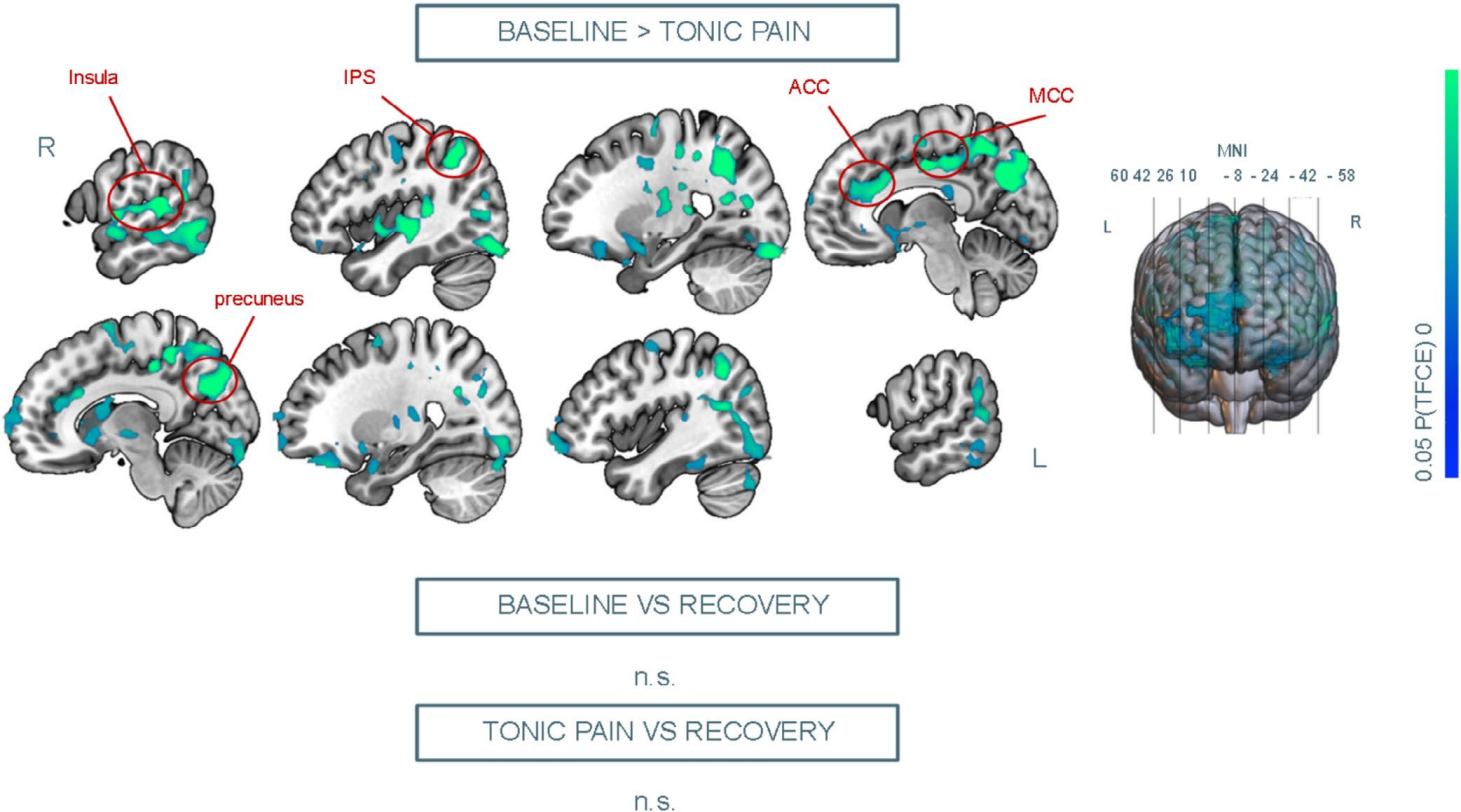
Seed‐based connectivity results. Significant map depicts decreases in functional connectivity (FC) with the seed (i.e., PAG) during the tonic pain condition compared to baseline. Results are significant following threshold‐free cluster enhancement (TFCE) correction at alpha 0.05. ACC = anterior cingulate cortex; IPS = intraparietal sulcus; L = left; MCC = midcingulate cortex; MNI = Montreal Neurological Institute; R = right.

Group analysis revealed that FC between the PAG and widespread cortical and subcortical areas was reduced during tonic pain compared to baseline. Areas involved included the right insula, the intraparietal sulcus (IPS), the ventral ACC, the midcingulate cortex (MCC) and the precuneus and the occipital cortex (Figure 5). We observed no significant FC differences across other contrasts of interest. We also observed no significant correlation between FC of the PAG and the rest of the brain during tonic pain and subjective average NRS scores.

### 3.4 DCM Results

**FIGURE 6:**
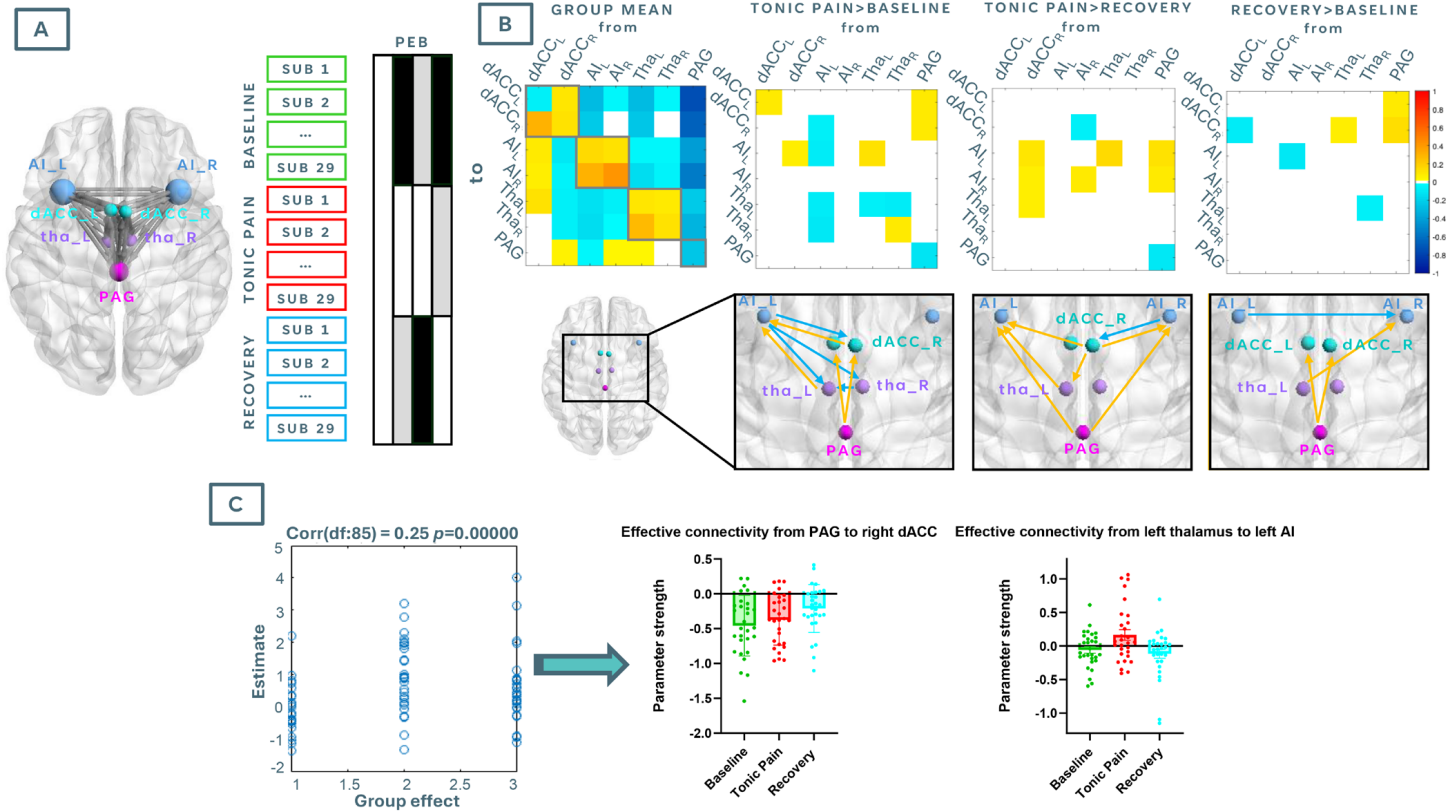
DCM results summary. (A) From left to right: Locations of seven nodes comprising the descending pain control pathway used for spDCM analysis. Due to the exploratory nature of the analysis, a fully connected model was initially described. (B) Results from greedy search through nested models; depiction of parametric empirical Bayes (PEB) design matrix, comprising scans from all participants across the three experimental conditions (‘Baseline’, ‘Tonic Pain’ and ‘Recovery’). Matrix columns reflect a group mean, and pairwise comparisons were specified (white colours indicate contrast loads = 1, grey colours indicate contrast loads = 0, black indicates contrast loads = −1). (B) Top row shows a depiction of connections with posterior probabilities greater than 0.75 across each contrast of interest. Off‐diagonal positive numbers (represented by yellow arrows on the corresponding bottom diagram) represent excitatory connections (*from* locations indicated by rows and *to* locations indicated by columns), and off‐diagonal negative numbers (represented by blue arrows on the corresponding bottom diagram) represent inhibitory connections. (C) From left to right: Leave‐one‐out cross‐validation (LOOCV) results. Parameters from connections with posterior probabilities greater than 0.9 for pairwise comparison results were used to predict the left‐out condition. The correlation between the predicted experimental condition and the actual condition, *r* is the correlation coefficient; connectivity strength across conditions from connections included in LOOCV. AI_L = left anterior insula; AI_R = right anterior insula; dACC_L = left dorsal anterior cingulate cortex; dACC_R = right dorsal anterior cingulate cortex; tha_L = left thalamus; tha_R = right thalamus; PAG = periaqueductal grey.

Individual models per subject and condition achieved acceptable data fit, with percentages of variance explained ranging from 83.88 to 94.89. For PEB results, only parameters for connections showing a posterior probability (*pp*; i.e., probability of the connection being present vs. being absent) greater than 75% are reported and shown in Figure 6.

#### 3.4.1 Differences in Effective Connectivity Across Conditions

Compared to baseline, effective connectivity during tonic pain was characterised by top‐down inhibitory connections from the left AI to the right dACC and the thalamus bilaterally, as well as inhibitory influence within the thalamus from right to left ROIs. We also observed bottom‐up excitatory connections from the PAG to the dACC bilaterally, as well as from the left thalamus and the left dACC to the left AI. Within‐ROI connectivity strength reflected higher self‐modulation of the left dACC and right thalamus and lower self‐modulation of the left AI and PAG during tonic pain than at baseline.

Effective connectivity during tonic pain compared to recovery was characterised by bottom‐up excitatory effective connectivity parameters, specifically from the PAG and from the right dACC to the AI bilaterally, and from the left thalamus to the left AI. We also observed distinct top‐down connections, such as inhibitory influence from right AI to right dACC and excitatory influence from the right dACC to the left thalamus. Only the right AI displayed higher self‐modulation during tonic pain than during recovery, together with lower self‐modulation of the PAG.

Finally, effective connectivity results during the recovery condition compared to the baseline condition displayed once again bottom‐up excitatory activity from the PAG to the dACC bilaterally, along with excitatory activity from the left thalamus to the right AI. We also observed an inhibitory connection from the left AI to the right AI, as well as lower self‐modulation of the left AI.

#### 3.4.2 Leave‐One‐Out Cross‐Validation (LOOCV)

For validation of our spDCM model, we assessed whether experimental conditions could be predicted based on differences in connectivity strength across parameters showing a *pp* > 0.9 in at least one of the contrasts of interest. These connections were from PAG to right dACC and from left thalamus to left AI. LOOCV analysis indicated that these connectivity parameters could successfully predict experimental conditions, with Pearson's correlation between LOO matrix and the predicted condition equal to 0.25 (*p* < 0.001) (Figure 6c). Plotting of parameters of interest during LOOCV showed that inhibitory connectivity strength from the PAG to the right dACC progressively decreased across conditions, whereas effective connectivity from the left thalamus to the left AI hovered around zero during Baseline, became on average more excitatory during Tonic Pain, and switched back towards more inhibitory during Recovery.

## 4 Discussion

In this study, we employed a novel strategy to carry out rs‐fMRI assessments in healthy individuals before, during and following prolonged tonic noxious cold stimulation, via immersion of the hand in cold gelled water, mimicking the time course of pain observed in CPT studies. Overall, our experimental paradigm successfully induced varying levels of pain across individuals. Fully exploratory ICA analysis revealed that during tonic pain, there was a widespread engagement of areas of the descending pain control network, alongside frontal and sensorimotor engagement that extended across 6 min following stimulation. Results from seed‐based connectivity analysis of the PAG with the rest of the brain reflected a decrease in FC with similar areas within this descending pain control network during tonic pain compared to baseline, but not during recovery. Results from DCM analysis within key nodes of the ascending and descending pain pathways revealed that compared to baseline, effective connectivity patterns during the tonic pain conditions were likely to be characterised by top‐down inhibitory influences from the left AI and more excitatory influences bottom‐up from PAG and thalamus, the latter ones being also similar during recovery compared to baseline. In this section, we discuss the potential mechanisms underlying tonic cold pain according to these results.

At group level, we successfully elicited mild‐to‐moderate tonic cold pain during 6 min inside the MRI scanner. Continuous VAS data during analogous stimulation outside of the scanner revealed that subjective pain ratings peaked between 2 and 3 min of noxious stimulation, reducing then to a lower and steady pain sensation. These results are completely consistent with earlier characterisation of responses to the CPT across minutes (Chang et al. 2002; Devoize et al. 2016; Eccleston 1995), albeit some variability (Shao et al. 2012), suggesting that immersion of the hand in gelled cold water can effectively replicate CPT‐like responses. While we did not collect continuous VAS ratings inside the MR scanner, as this would have confounded the resting state nature of the paradigm, the average NRS ratings provided by participants following the tonic pain scan did not significantly differ from average VAS ratings. This result points towards successful reproducibility ratings across both hands; however, future investigations may elucidate the test–retest reliability of tonic cold pain ratings via gelled cold water.

Crucially, ICA results indicated that our tonic cold paradigm induced temporally synchronised activity of dACC, midcingulate cortex, AI bilaterally, basal ganglia, thalamus, amygdala, brainstem (including pons, PAG and rostroventral medulla (RVM)) and cerebellum. These patterns emerged independently of subjective pain ratings, as ICA was performed in a fully data‐driven manner without incorporating behavioural regressors, thereby evidencing spontaneous engagement of areas across cortical and subcortical descending pain control pathways (Bannister 2019; Bannister and Dickenson 2017; Chen and Heinricher 2019). This was accompanied by an engagement of widespread sensory and motor areas; while somatosensory activation during stimulation can usually be attributed to processing of discriminative aspects of noxious stimulation, such as location, quality and intensity (Oshiro et al. 2009; Sun et al. 2023), activation of motor regions during tonic noxious stimulation has been suggested to arise as a result of self‐protective aversive reactions during tonic muscle pain (Rossi et al. 2003). Somatosensory alongside prefrontal engagement was also observed during tonic pain, consistent with previous findings within experimental pain conditions. Activity in these regions is thought to support broader sensorimotor integration, including attention, affective‐motivational processing, cognitive control and preparation for adaptive behavioural responses to sustained noxious input (Dancey et al. 2016; Garcia‐Larrea and Peyron 2013; Misra et al. 2017; Nickel et al. 2020; Perini and Bergstrand 2013). Consequently, reappraisal of sensorimotor integration following prolonged tonic cold pain could explain why both prefrontal and sensorimotor independent components persisted during the recovery phase. It is worth noting that no direct statistical comparisons were conducted across ICA maps between conditions. As ICA is inherently data‐driven, it does not ensure functional correspondence between networks, especially when experimental manipulations can induce significant reorganisation of the underlying networks. Visual inspection of our data clearly indicates such reorganisation, and attempting forced comparisons could misrepresent these complex dynamics (Laird et al. 2011). Another intriguing finding from our ICA analysis was the increased extension of the brainstem‐cerebellar component during tonic noxious stimulation compared to the other conditions. This is consistent with positron emission tomography (PET) findings during the CPT, where brainstem activation was related to initial responses to cold noxious stimulation and changes in galvanic skin responses, suggesting its involvement in sympathetic responses during noxious cold (Petrovic et al. 2004). Increases in regional cerebral blood flow (rCBF) have also been observed across the descending pain control pathway and cerebellum during the CPT (Casey et al. 1996). Increasing evidence suggests that the cerebellum plays a crucial modulatory role during nociception (Moulton et al. 2010), temperature detection (Zunhammer et al. 2011) and top‐down pain modulation via its communication with sensorimotor, executive, reward and subcortical structures such as the amygdala, hippocampus and thalamus, through the brainstem (Li et al. 2024). Taken together, these findings indicate that our cold pain paradigm successfully elicited functional connectivity patterns consistent with those observed in studies of perceived pain generally and the CPT specifically. Notably, these results emerged from an entirely data‐driven approach, such as ICA, which minimises bias in the identification of neural networks. This underscores the utility of the current paradigm as a robust method for inducing CPT‐like pain neural signatures during fMRI in healthy individuals, offering a viable platform for investigating pain processing and connectivity dynamics. While we did not collect continuous physiological measures during fMRI or continuous VAS scores during tonic pain in the same hand stimulated in the scanner, future iterations of this experiment may explore how emerging ICA components relate to concurrent individual temporal variations in pain in the same body location or objective measures related to pain perception processing (such as hear rate variability) during scanning.

Our seed‐based connectivity analysis revealed a disengagement of the PAG from an array of cortical areas, including right insula, IPS, ACC, midcingulate cortex, precuneus and occipital cortex, during tonic pain compared to baseline, partly overlapping with previous findings in healthy volunteers (Makovac et al. 2020). This was likely due to a shift towards an engagement with downstream interactions with the brainstem and spinal cord to inhibit cold noxious input (Leith et al. 2010; Makovac et al. 2021; Xin et al. 1997). While previous findings showed correlations between PAG activation during cold tonic (La Cesa et al. 2014) as well as evoked (Mohr et al. 2008) pain and subjective ratings, FC of the PAG did not correlate with individual subjective pain scores; this result suggests that PAG‐led pain modulation might rely more on the action of top‐down PAG‐RVM‐spinal opioidergic pathways than on the extent of PAG‐brain synchronisation, which has been suggested to be more closely related to autonomic regulation during pain (Hohenschurz‐Schmidt et al. 2020; Makovac et al. 2021). Nevertheless, sympathetic regulation may indirectly modulate PAG‐RVM inhibition through stress‐induced analgesia (Lau and Vaughan 2014). Moreover, PAG‐cortex disengagement was not observed during the Recovery condition compared to the Tonic Pain condition, hinting towards sustained modulatory action following withdrawal of tonic cold stimuli.

In fact, our spDCM results revealed that effective connectivity patterns during the tonic pain condition, compared to baseline, were most likely characterised by more excitatory influences from the PAG to the dACC bilaterally and from the thalamus to the AI, a pattern that persisted during recovery compared to baseline. The PAG is a central hub for pain modulation, acting top‐down and as a bottom‐up relay of pain responses and nociceptive drive, respectively (for a review, see Linnman et al. (2012)). During tonic pain, it is reasonable to argue that the PAG might amplify excitatory input to the dACC to enhance the perception and emotional salience and aversion of pain, encouraging protective or adaptive behaviours (Zhang et al. 2024). This would also explain why the tonic pain condition was characterised by heightened excitatory influences from the PAG to the AI than during recovery. Nevertheless, bottom‐up PAG‐dACC excitatory influence persisted during recovery compared to baseline, which suggests recalibration of both sensory and affective circuits (Hohenschurz‐Schmidt et al. 2020; Zhang et al. 2024). The PAG might sustain excitatory inputs to the dACC during early recovery to process and resolve the lingering affective and evaluative aspects of pain. Similarly, our results suggest that the left thalamus acted as a relay of nociceptive drive to the left AI during tonic pain, probably resulting from the activation of spinothalamic–cortical pathways (Benarroch and Benarroch 2021; Gélébart et al. 2022; Groh et al. 2017; Jalon et al. 2023). Our LOOCV results showed that this influence switched towards inhibition during the Recovery scan, arguably reflecting an adaptive action to recalibrate the pain network and reset to baseline. Conversely, compared to Baseline, effective connectivity during tonic pain was likely characterised by top‐down inhibitory action of the AI on the dACC and thalamus bilaterally, which might reflect a regulatory attempt to downscale the dACC's activity and a top‐down mechanism to prevent excessive engagement with the aversive aspects of pain (Alonso‐Matielo et al. 2023; Ossipov et al. 2010), which could otherwise lead to heightened distress or maladaptive behavioural responses (Buchmann et al. 2021).

In general, LOOCV revealed that effective connectivity of the PAG‐dACC and thalamus–AI pathways could successfully predict experimental conditions, highlighting the state‐specific modulation of pain‐related networks. These findings underscore the mechanistic roles of ascending pathways in processing the sensory, affective and regulatory components of pain and its resolution. The predictive accuracy of these connections positions them as potential biomarkers for characterising the dynamics of pain states and transitions, with implications for personalised pain management strategies.

A limitation of this study is the relatively small sample size of 29 participants. While a larger sample could enhance the robustness and generalisability of the findings, the use of advanced fMRI acquisition, preprocessing and analyses aimed to maximise sensitivity to subtle effects and provide novel, proof‐of‐concept evidence that allows a priori power calculations for follow‐up studies. It is also worth emphasising that although the short scan duration can be interpreted as a limitation, the short TR (1.5 s) and multiband and multiecho acquisition aimed to maximise signal‐to‐noise ratio despite the brief scanning period, balancing optimal data quality with experimental considerations (e.g., avoiding intolerable tonic pain due to scan length or habituation effects). Finally, it is important to acknowledge that while we took carefully considered steps to ensure precise targeting of our ROIs for signal extraction, the limited spatial resolution inherent to fMRI, alongside signal interpolation via data normalisation and smoothing, which was necessary to ensure a common space for all analysis approaches and optimal signal‐to‐noise ratio, could have affected the specificity of our seeds, especially in the case of small regions like the PAG.

## 5 Conclusion

In conclusion, this study establishes the cold gelled‐water paradigm as a potentially viable in‐scanner alternative to the CPT, effectively replicating its neurophysiological signatures and enabling the study of tonic pain in healthy individuals. We revealed distinct neural dynamics underpinning CPT‐like pain, including PAG‐driven excitatory inputs to dACC and AI that amplified pain salience, alongside AI‐mediated inhibitory control over dACC and thalamus to regulate sustained nociceptive and affective responses. Each analysis method provided unique insights: ICA captured large‐scale network dynamics, seed‐based connectivity revealed specific functional reorganisations of the PAG and DCM clarified directional influences within pain pathways. While future work directly comparing this method with the CPT will be important for further validation, these findings underscore the paradigm's utility for probing pain modulation and transitions, offering a robust platform for studying adaptive and maladaptive neural responses to pain.

## Supporting information


Data S1.

## Data Availability

The data that support the findings of this study are available on request from the corresponding author. The data are not publicly available due to privacy or ethical restrictions.
