# Supplementary material for: A Novel Investigation of an In‐Scanner Alternative to the Cold Pressor Test in Healthy Individuals
Source: Hum Brain Mapp. 2025 Jul 25;46(11):e70291. doi: 10.1002/hbm.70291 (PMC12290945; doi:10.1002/hbm.70291)
Supplement: Supplementary file 1 — Data S1. [file HBM-46-e70291-s001.docx]

**Supplementary Information**

1. **ROI definition**

| **ROI** | **BNA atlas labels** | **MNI coordinates** | | **Radius size** |
| --- | --- | --- | --- | --- |
|  |  | **Left** | **Right** |  |
| dorsal ACC | A24cd, caudodorsal area 24 | -5, 7, 37 | 4, 6, 38 | 4mm |
| AI | dIa, dorsal agranular insula | -34, 18, 1 | 36, 18, 1 | 8mm |
| thalamus | mPFtha, medial pre-frontal thalamus | -7, -12, 5 | 7, -11, 6 | 4mm |

**Supplementary table 1.** ROI definition details. ACC = anterior cingulate cortex; AI = anterior insula; BNA = Brainnetome Atlas; MNI = Montreal Neurological Institute


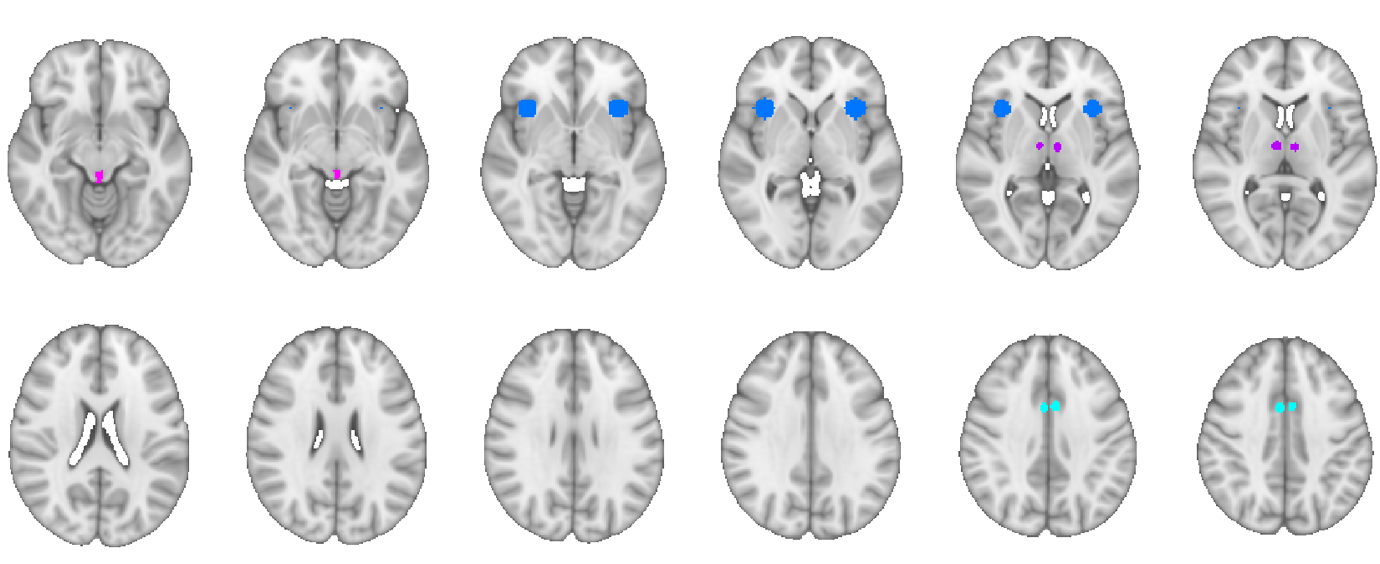


**Supplementary Figure 1.** Depiction of dACC (light blue), AI (dark blue), thalamus (purple) and PAG (pink) ROIs.

1. **ICA components 5-10**


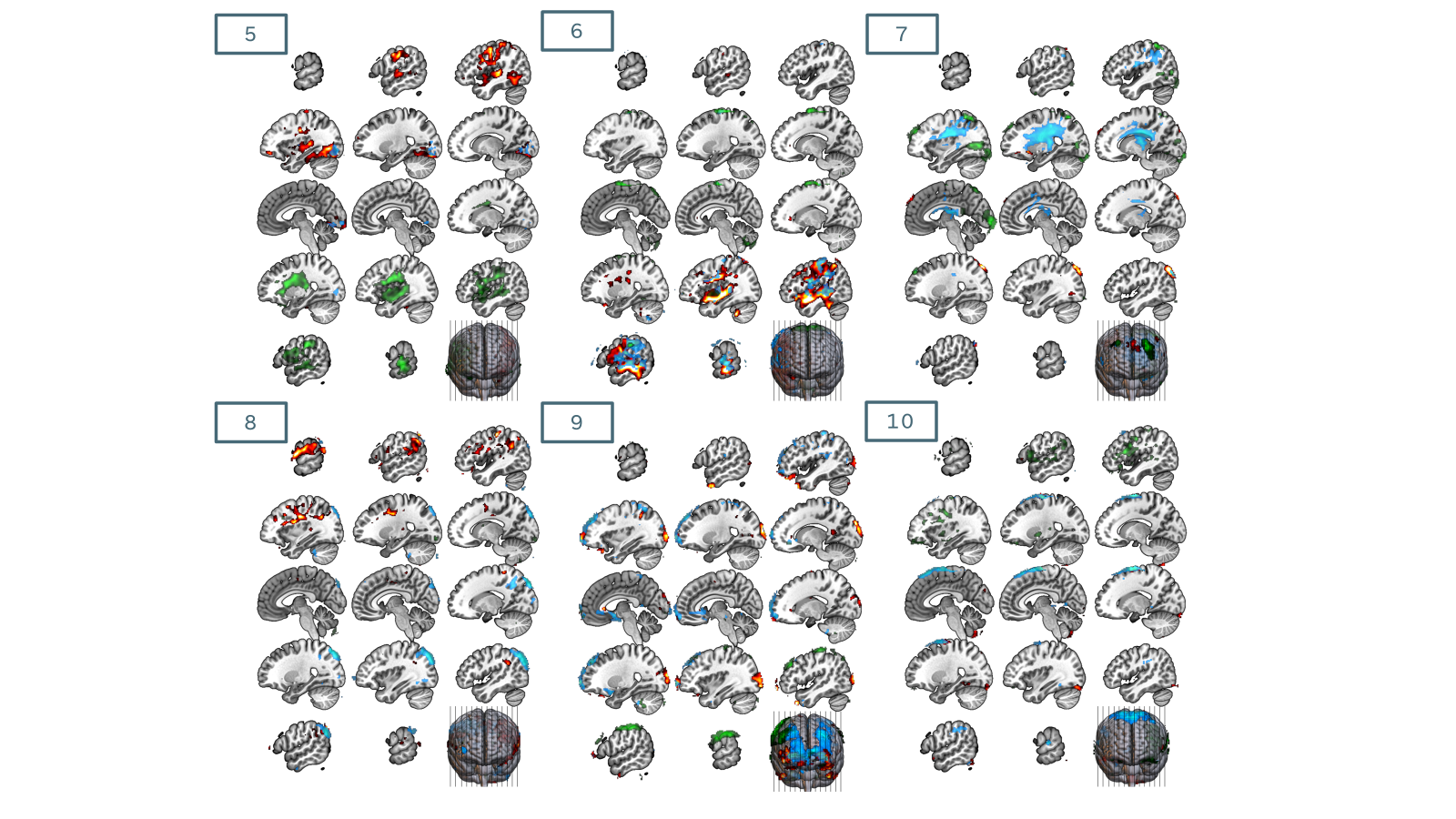


**Supplementary figure 2. Components 5-10 from ICA analysis.**
